# Microbiome–mycotoxin interactions and probiotic strategies: implications for gut health and cancer

**DOI:** 10.3389/fnut.2026.1783295

**Published:** 2026-02-25

**Authors:** Alice N. Mafe, Dietrich Büsselberg

**Affiliations:** 1 Faculty of Sciences, Department of Biological Sciences, Taraba State University, Jalingo, Nigeria; 2 Faculty of Medicine, Department of Physiology and Biophysics, Weill Cornell Medicine-Qatar, Education City, Qatar Foundation, Doha, Qatar

**Keywords:** bioactive microorganisms, biotherapeutic interventions, carcinogenic signaling, epithelial barrier function, host–microbe modulation, intestinal dysbiosis, microbial detoxificationenzymes, xenobiotic biotransformation

## Abstract

This structured, hypothesis-driven narrative review examines how mycotoxins, pervasive food contaminants, disrupt intestinal microbial balance, epithelial barrier integrity, xenobiotic metabolism, and carcinogenic signaling. Emerging evidence indicates that bidirectional interactions between the gut microbiome and mycotoxins modulate these effects, with microbial detoxification enzymes influencing toxin metabolism, immune responses, and epithelial resilience. However, the mechanistic understanding of microbiome–mycotoxin interplay remains incomplete, particularly regarding enzymatic pathways, microbial metabolites, and cancer-associated signaling. This review synthesizes recent (2016–2025) mechanistic studies on gut microbiota–mediated mycotoxin biotransformation, enzymatic detoxification, and probiotic interventions as strategies to mitigate mycotoxin-induced gut and cancer-related damage, focusing on key dietary toxins such as aflatoxin B₁, deoxynivalenol, zearalenone, ochratoxin A, fumonisins, and patulin. Evidence indicates that microbial enzymes, including de-epoxidases, lactonases, and reductases, contribute to mycotoxin biotransformation, while probiotics can enhance epithelial barrier function, restore microbial ecosystem balance, and modulate immune responses through toxin binding, competitive exclusion, and anti-inflammatory actions. The review further highlights the strain-specific nature of detoxification, the impact of mycotoxin-induced dysbiosis on short-chain fatty acid production and inflammation, and the modulation of cancer-related pathways including NF-κB, STAT3, and IL-6. Finally, it provides an integrated framework linking microbial mechanisms, bioactive microorganisms, and regulatory considerations, identifies critical knowledge gaps, and outlines mechanistically informed probiotic strategies for mitigating mycotoxin exposure and its associated health risks.

## Graphical abstract

**Figure:**
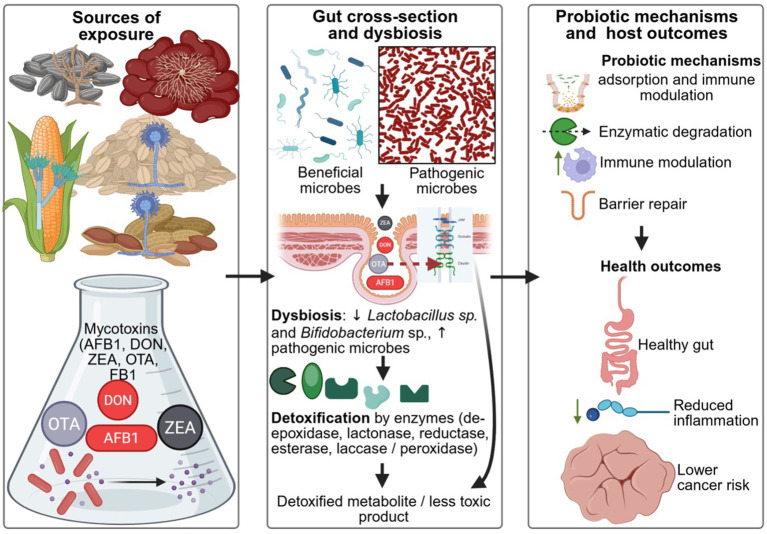
Graphical abstract created in BioRender. Busselberg, D. (2025). https://app.biorender.com/illustrations/6927c7314322ab4a209c929f.

## 1 Introduction

Mycotoxins are a diverse group of secondary metabolites produced by fungi such as *Aspergillus* sp., *Penicillium* sp., and *Fusarium* sp. (1). Their widespread contamination of food products results in ongoing human exposure, particularly in low- and middle-income regions where monitoring systems are limited (2). In parallel, scientific interest in the gut microbiome has grown, revealing its central role in regulating immune function, metabolic processes, and cancer development (3). Recent studies show that gut microbes actively influence mycotoxin metabolism (4–6), while the toxins themselves alter microbial composition, forming a bidirectional interaction with significant health implications (7–9). Although substantial research exists, our understanding of the mechanistic interplay between mycotoxins and the gut microbiome, particularly with respect to gut health and carcinogenesis, remains incomplete. This review is driven by several key motivations like the increasing incidence of colorectal and hepatocellular cancers associated with chronic toxin exposure, the growing reliance on natural detoxification approaches such as probiotics, and the absence of reviews that effectively integrate microbial ecology, enzymatic pathways, and cancer-related mechanisms.

Critical gaps and controversies continue to challenge the field. Conflicting evidence persists regarding whether specific microbiota detoxify (10–12) or conversely activate specific mycotoxins (13–15). Many existing reviews fail to explore detoxification at the enzymatic level or address strain-specific mechanisms. Furthermore, only a few studies have attempted to link microbial ecological shifts to inflammation-driven cancer pathways. There is also a lack of comprehensive syntheses that bridge findings from *in vitro* experiments, animal models, and clinical data. Earlier reviews often focus on isolated aspects of individual toxins, probiotics, or cancer outcomes without examining their interconnections. Many lack up-to-date data, offer limited mechanistic insight, or overlook emerging advances such as engineered probiotics and omics-based analytical tools. The present review seeks to overcome these limitations by providing a more current, mechanistically detailed, and innovation-focused perspective.

This review is deliberately focused on elucidating gut microbiota–mediated enzymatic and metabolic mechanisms that influence the intestinal toxicity and carcinogenic potential of major dietary mycotoxins. The primary objective is to critically synthesize evidence on how microbial xenobiotic biotransformation, detoxification enzymes, and metabolic activation pathways modulate mycotoxin bioavailability, epithelial barrier integrity, inflammation, and cancer-related signaling within the gastrointestinal tract. A secondary objective is to evaluate strain-specific probiotic strategies that mitigate mycotoxin-induced damage through well-defined mechanisms, including toxin binding, enzymatic biotransformation, and host–microbe immune modulation. These probiotic interventions are discussed in the context of mechanistic efficacy rather than broad health claims. Additional topics such as emerging technological tools, regulatory considerations, and future research directions are included only as contextual extensions to support interpretation and translational relevance and are not treated as independent research questions. Veterinary applications, extra-intestinal toxic effects, and non-mycotoxin microbial contaminants are explicitly excluded. By narrowing its focus to microbiome-driven mechanisms and probiotic-mediated mitigation within the gut, this review provides a coherent, mechanistically grounded, and search-strategy-aligned synthesis of current knowledge, while offering a unified framework linking microbial ecology to toxin-induced intestinal and cancer-associated outcomes.

## 2 Methodology

A comprehensive literature search was conducted across several scientific databases, including ScienceDirect (https://www.sciencedirect.com), Google Scholar (https://scholar.google.com), PubMed (https://pubmed.ncbi.nlm.nih.gov), and SciELO (https://scielo.org). These platforms were chosen for their extensive coverage of studies in microbiology, toxicology, oncology, and food safety, providing a strong foundation for exploring microbiome–mycotoxin interactions. This article is a narrative review that adopts a selective, hypothesis-driven literature synthesis rather than a formal systematic review approach. Accordingly, the literature search was designed to capture recent and mechanistically informative studies relevant to microbiome–mycotoxin interactions, rather than to provide an exhaustive or statistically pooled evaluation of all available evidence. Eligible publications were selected based on the following criteria: written in English, published between 2016 and 2025, and focused on topics such as microbiome–mycotoxin interactions, enzymatic detoxification pathways, gut health, or cancer-related mechanisms. The review period was restricted to studies published between 2016 and 2025 to emphasize recent mechanistic advances, omics-driven insights, and probiotic innovations; however, this temporal focus may limit coverage of earlier foundational studies, which are acknowledged as important but beyond the primary scope of this review. Research articles, review papers, theses, and relevant e-books were considered for inclusion to allow broad contextual synthesis appropriate for a narrative review. To ensure scientific relevance and quality, studies were excluded if they were written in languages other than English, were unrelated to the core themes of this review, or lacked sufficient methodological rigor or mechanistic depth. The literature search was guided by a defined set of core thematic keywords aligned with the scope of this review, including intestinal dysbiosis, xenobiotic biotransformation, microbial detoxification enzymes, epithelial barrier function, carcinogenic signaling, bioactive microorganisms, host–microbe modulation, and biotherapeutic interventions. Duplicate entries were removed and managed using Mendeley Desktop reference management software. When review articles were included, only primary experimental data not duplicated elsewhere were considered. Original studies cited within reviews were cross-checked to avoid double-counting of evidence. Titles and abstracts underwent preliminary screening, followed by full-text reviews to assess suitability. Titles, abstracts, and full texts were screened collaboratively by the two authors to assess relevance, methodological clarity, and alignment with the objectives of this narrative review. Any differences in interpretation were resolved through discussion and consensus. Ultimately, only studies that demonstrated strong methodological quality and clear relevance to microbiome–mycotoxin interactions, enzymatic mechanisms, probiotics, or cancer pathways were included in the final analysis. As a narrative review, this synthesis is inherently limited in its ability to quantitatively account for the real-world complexity of exposure, including chronic low-dose intake and the co-occurrence of multiple mycotoxins in habitual diets. These constraints were considered during the interpretation of findings, and conclusions were framed cautiously to avoid overgeneralization beyond the available experimental evidence.

### 2.1 Quality appraisal and bias considerations

As this work is a narrative review, formal systematic scoring or risk-of-bias tools were not applied. Instead, the included literature was critically evaluated to prioritize studies with clear experimental models, explicit identification and dosing of specific mycotoxins, strain-level microbial characterization, and reporting of mechanistic endpoints relevant to gut health and carcinogenesis. Studies lacking sufficient methodological clarity or mechanistic relevance were interpreted cautiously and used primarily for contextual discussion. For tables summarizing probiotic–mycotoxin interactions (e.g., Table 1), the “indicative strength of evidence” reflects the consistency, biological plausibility, and study type (*in vitro*, *in vivo*, or clinical) reported in the literature, rather than a formal systematic grading. Limitations related to analytical sensitivity especially the detection of low mycotoxin concentrations in fecal or intestinal samples, and variability in probiotic survival, bioavailability, and functional activity *in vivo,* were explicitly considered during evidence appraisal and contributed to conservative assignment of indicative strength of evidence where appropriate. While this approach may introduce selection bias inherent to narrative syntheses, transparency in scope definition and cautious phrasing of conclusions were used to minimize overinterpretation. The operational thresholds used to assign ‘indicative strength of evidence’ (strong, moderate, limited, emerging) for probiotic–mycotoxin interactions are provided in Supplementary Table S2 which ensures transparency and reproducibility of evidence classification.

**Table 1:** Probiotic strains and their mechanisms of mycotoxin detoxification.

Probiotic species/strain (example)	Detoxification mechanism (binding, enzymatic degradation, immune modulation, competitive exclusion)	Toxin targeted (examples)	Evidence type (*in vitro/in vivo* /clinical)	Indicative strength of evidence (operationalized)	References
*Lactobacillus rhamnosus* (e.g., GG)	Cell-wall binding; immune modulation	AFB₁, OTA	*In vitro, in vivo* (animal)	Strong = ≥2 *in vivo* + consistent mechanism	(134–136)
*Lactobacillus plantarum* (various strains)	Binding; enzymatic transformation; competitive exclusion	AFB₁, DON, ZEA, OTA	*In vitro*, some selected *in vivo*	Moderate = multiple *in vitro* + limited *in vivo*	(137)
*Lactobacillus acidophilus*	Surface adsorption (binding); modulation of gut immunity	AFB₁, OTA	*In vitro*, limited *in vivo*	Moderate	(138, 139)
*Lactobacillus casei*	Binding; competitive exclusion	AFB₁, ZEA	*In vitro*, some animal studies	Moderate	(140, 141)
*Lactococcus lactis*	Binding; possible enzymatic activity (strain-dependent)	AFB₁, DON	*In vitro* only	Limited = *in vitro* only or inconsistent results	(142, 143)
*Bifidobacterium longum*	Binding; gut barrier/immune modulation	AFB₁, OTA	*In vitro*, some *in vivo*	Moderate	(144–146)
*Bifidobacterium bifidum*	Surface adsorption; immune modulation	AFB₁, ZEA	*In vitro* only	Limited	(147, 148)
*Saccharomyces cerevisiae/Saccharomyces boulardii*	Cell-wall (glucomannan/β-glucan) adsorption; competitive exclusion; immune modulation	AFB₁, OTA, ZEA	*In vitro*, *in vivo* (animal); some clinical probiotic safety/efficacy data	Strong	(149, 150)
*Bacillus subtilis* (selected strains)	Enzymatic degradation (extracellular enzymes); binding	FB₁, ZEA, DON (strain-dependent)	*In vitro*, several animal studies	Moderate	(137, 151, 152)
*Enterococcus faecium* (selected strains)	Binding; competitive exclusion	AFB₁, DON	*In vitro*, limited *in vivo*	Limited	(153–155)
*Pediococcus pentosaceus*	Binding; fermentation-associated detoxification	AFB₁, DON	*In vitro* only	Limited / Emerging	(156)
Mixed commercial probiotic consortia (defined blends)	Combined mechanisms: binding, enzymatic activity, immune modulation	Multiple mycotoxins (broad claims)	*In vitro*, some *in vivo*; rare clinical	Emerging / Variable	(157, 158)

1. Indicative strength of evidence is operationalized as follows: Strong = ≥2 *in vivo* studies with consistent detoxification mechanism; Moderate = multiple *in vitro* studies with limited *in vivo* evidence; Limited = *in vitro* only or inconsistent results; Emerging/variable = preliminary evidence with partial or inconsistent mechanistic data.

2. Evidence type is explicitly distinguished as *in vitro*, *in vivo* (animal), or clinical.

AFB₁, aflatoxin B₁; OTA, ochratoxin A; DON, deoxynivalenol; ZEA, zearalenone; FB₁, fumonisin B₁.

### 2.2 Literature search strategy and study selection

**Figure 1:**
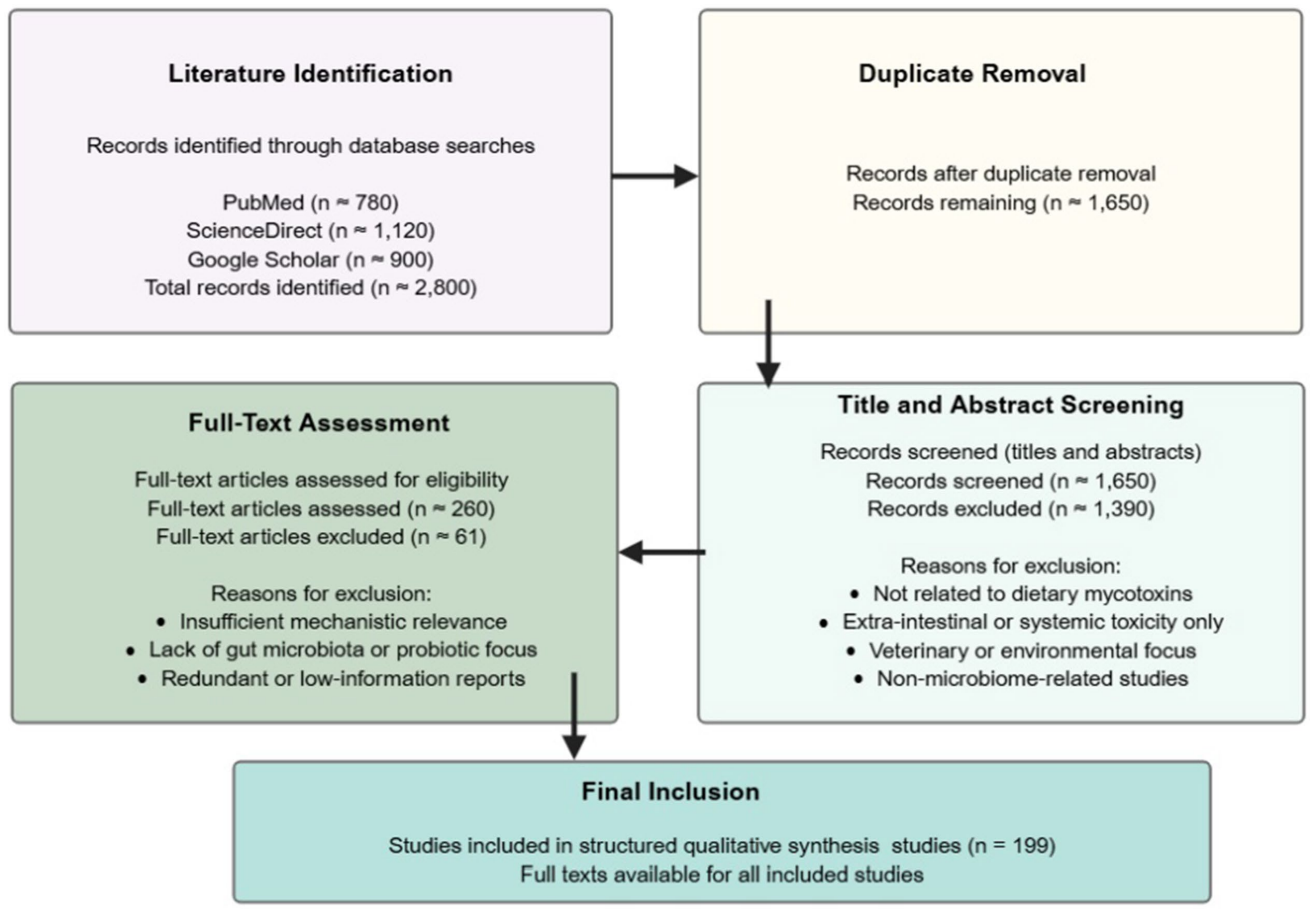
Literature selection workflow for the structured narrative synthesis. Created in BioRender. Busselberg, D. (2025). https://app.biorender.com/illustrations/6970acbb12b757d946b7a3f0. Flow diagram illustrating the literature identification, screening, and study inclusion process applied in this structured, hypothesis-driven narrative literature synthesis. The workflow summarizes database searching, duplicate removal, relevance screening, full-text assessment, and final inclusion of studies focusing on gut microbiota–mediated modulation of dietary mycotoxin toxicity and probiotic-based mitigation mechanisms.

This review was conducted as a structured, hypothesis-driven narrative literature synthesis, guided by predefined mechanistic questions examining how gut microbiota–mediated enzymatic and metabolic processes influence the intestinal toxicity and carcinogenic potential of dietary mycotoxins, and how probiotic-based interventions mitigate these effects within the gastrointestinal tract. A comprehensive literature search was conducted across PubMed, ScienceDirect, and Google Scholar, covering peer-reviewed articles published through December 2025. Searches were performed using predefined keyword blocks combined with Boolean operators to capture relevant studies. Mycotoxin-related terms such as mycotoxin, mycotoxins, aflatoxin, aflatoxin B1, deoxynivalenol (DON), zearalenone, ochratoxin A (OTA), fumonisin (FB), T-2 toxin, trichothecene, and patulin (PAT). Gut microbiota and intestinal context terms included gut microbiota, gut microbiome, intestinal microbiota, intestinal microflora, intestinal epithelium, intestinal barrier, tight junctions, and commensal bacteria. Microbial metabolism and detoxification terms included xenobiotic metabolism, microbial biotransformation, detoxification enzymes, mycotoxin degradation, microbial enzymatic detoxification, biotransformation pathways, enzymatic degradation, and cytochrome P450. Probiotics and mitigation strategies included probiotics, lactic acid bacteria, *Lactobacillus*, *Lactiplantibacillus*, *Bifidobacterium*, *Bacillus*, strain-specific interventions, toxin binding, and bioadsorption. Intestinal and cancer-related outcomes included intestinal toxicity, intestinal barrier, epithelial permeability, inflammation, oxidative stress, carcinogenesis, cancer signaling, NF-κB, Wnt/*β*-catenin, MAPK, apoptosis, epigenetic modulation, and colorectal cancer. Boolean operators were applied as follows: (mycotoxin terms) AND (gut microbiota terms) AND (detoxification or probiotic terms), with additional outcome-specific filters applied where relevant. The literature search identified approximately 780 records from PubMed, 1,120 from ScienceDirect, and 900 from Google Scholar, yielding a total of approximately 2,800 records across databases. After removal of duplicate entries, approximately 1,650 unique records remained and were screened based on titles and abstracts. Studies were excluded if they did not involve dietary mycotoxins, lacked relevance to the gastrointestinal tract, focused exclusively on veterinary, environmental, or extra-intestinal outcomes, or addressed non-mycotoxin microbial contaminants. Following this screening, approximately 260 full-text articles were assessed for eligibility. Ultimately, 199 peer-reviewed studies were included in the final structured qualitative synthesis, comprising 72 studies from PubMed, 64 from ScienceDirect, and 63 from Google Scholar. Full-text versions were available for all included articles. Quantitative outputs of the literature search and study selection process are summarized in Supplementary Table S1, and an overview of the screening workflow is presented in Figure 1. Additionally, the rationale and thresholds for categorizing the strength of evidence for each included probiotic–mycotoxin study are detailed in Supplementary Table S2 and link the study design, *in vitro*/*in vivo* evidence, and mechanistic consistency to the assigned indicative strength.

## 3 Thematic and mechanistic sections

### 3.1 Microbiome alterations caused by mycotoxins

Mycotoxins significantly disrupt the composition and functional stability of the gut microbiome, leading to distinct patterns of dysbiosis (16). One of the most consistent effects is the depletion of beneficial commensal bacteria, particularly *Lactobacillus* sp. and *Bifidobacterium* species, which play vital roles in maintaining microbial balance, producing antimicrobial compounds, and supporting immune and metabolic functions (17). The decline of these organisms weakens mucosal defenses and favors the overgrowth of pathogenic or opportunistic taxa (18, 19). Mycotoxin exposure also impairs the production of short-chain fatty acids (SCFAs), notably butyrate, acetate, and propionate, by altering the activity of fermentative microbial communities (20, 21). Reduced SCFA availability can compromise epithelial energy supply, disrupt mucosal repair mechanisms, and diminish anti-inflammatory signaling (22–24). In addition, mycotoxins interfere with bile acid metabolism, shifting the balance between primary and secondary bile acids, which further influences microbial composition, nutrient absorption, and intestinal barrier stability (25, 26) together, these alterations undermine mucosal health, promote inflammatory conditions, and increase susceptibility to gastrointestinal dysfunction. For example, aflatoxin B₁ (AFB1) exposure is consistently associated with reductions in *Lactobacillus* sp. and *Bifidobacterium* sp. populations and decreased butyrate levels, whereas deoxynivalenol (DON) predominantly affects epithelial turnover and villus integrity. These and other toxin-specific microbial signatures are summarized in Table 2.

**Table 2:** Summary of Gut Microbiome Alterations Associated with Major Mycotoxins.

Mycotoxin	Altered microbial Taxa (↑/↓)	Affected metabolites	Gut barrier effects	References
Aflatoxin B1 (AFB1)	↓ *Lactobacillus* sp., ↓ *Bifidobacterium* sp., ↑ *Enterobacteriaceae*, ↑ *Clostridium* sp.	↓ SCFAs (butyrate, acetate), ↑ ammonia, and altered bile acids	Tight junction disruption (↓ occludin, ↓ claudin-1), increased intestinal permeability, epithelial inflammation	(159–161)
Fumonisin B1 (FB1)	↓ *Lactobacillus* sp., ↓ *Ruminococcaceae*, ↑ *Bacteroides*	Sphingolipid disruption, ↓ butyrate, ↑ pro-inflammatory metabolites	Impaired mucin layer, compromised tight junction proteins, increased LPS translocation	(162–164)
Zearalenone (ZEA)	↓ *Lactobacillus* sp., ↑ *Proteobacteria*, ↑ *Prevotella* sp.	Altered estrogen-like metabolites, ↓ SCFAs	Barrier weakening, inflammatory cytokine surge (IL-6, TNF-α), oxidative stress	(165–167)
Ochratoxin A (OTA)	↓ *Bacteroidetes*, ↓ *Firmicutes*, ↑ *Enterococcus* sp., ↑ *Clostridium* sp.	↓ SCFAs, ↑ reactive oxygen species, altered tryptophan metabolites	Mitochondrial stress in enterocytes, tight junction disruption, impaired nutrient absorption	(168–170)
Deoxynivalenol (DON)	↓ *Lactobacillus* sp., ↓ *Bifidobacterium*, ↑ *Escherichia* sp./*Shigella* sp., ↑ *Clostridiaceae*	↓ butyrate, ↑ lactate, altered bile acids	Villus atrophy, epithelial apoptosis, increased intestinal permeability	(171–173)
T-2 Toxin	↓ *Firmicutes*, ↓ *Lactobacillus* sp., ↑ *Proteobacteria*	↓ SCFAs, altered amino acid metabolites	Severe mucosal injury, villus damage, compromised immune signaling	(174–176)
Patulin	↓ *Lactobacillus* sp., ↑ *Enterobacter* sp.	↓ butyrate, ↑ oxidative metabolites	ROS-induced epithelial injury, disruption of tight junction proteins	(177–179)

### 3.2 Mechanisms of mycotoxin toxicity in the gut together

Mycotoxins exert harmful effects through several interconnected mechanisms that compromise intestinal integrity and host physiology (27, 28). A central process is the induction of oxidative stress, in which mycotoxins stimulate excessive production of reactive oxygen species (ROS), leading to lipid peroxidation, protein oxidation, and DNA damage (29, 30). Some mycotoxins like AFB1 is particularly associated with ROS generation and DNA damage, DON predominantly induces ribotoxic stress and oxidative stress, whereas zearalenone (ZEA) primarily disrupts estrogen-responsive signaling pathways (31–33), while simultaneously modulating cytochrome P450 (CYP450) pathways that affect detoxification and co-exposed compound metabolism (34, 35). DON and T-2 toxin predominantly induce ribotoxic stress and epithelial apoptosis, T-2 toxin disrupts intestinal mucin by activating the inositol-requiring enzyme 1 alpha (IRE1α) / X-box binding protein 1 (XBP1) endoplasmic reticulum (ER) stress pathway, which downregulates mucin 2 (MUC2) expression, alters mucus barrier integrity, promotes microbial–epithelial contact, and contributes to gut dysbiosis and pro-inflammatory cytokine induction (36). Zearalenone (ZEA) primarily disrupts estrogen-responsive signaling, whereas ochratoxin A (OTA) causes mitochondrial stress and impairs DNA repair. The gut epithelial barrier is a major target, as mycotoxins disrupt tight junction complexes by altering the expression and localization of key proteins such as occludin and claudins, thereby increasing intestinal permeability (37, 38). Immune dysregulation further contributes to toxicity, as mycotoxins can suppress protective immune responses while promoting pro-inflammatory cytokine production (39). Importantly, modulation of the gut microbiota can mitigate these effects; for example, *Lactobacillus rhamnosus* GG enhances butyrate production, which protects epithelial integrity and counteracts DON-induced toxicity (40). Through these combined, toxin-specific mechanisms, mycotoxins compromise gut function, enhance systemic exposure to harmful agents, and increase the risk of inflammation-driven diseases.

### 3.3 Enzymatic pathways and biotransformation mechanisms

**Figure 2:**
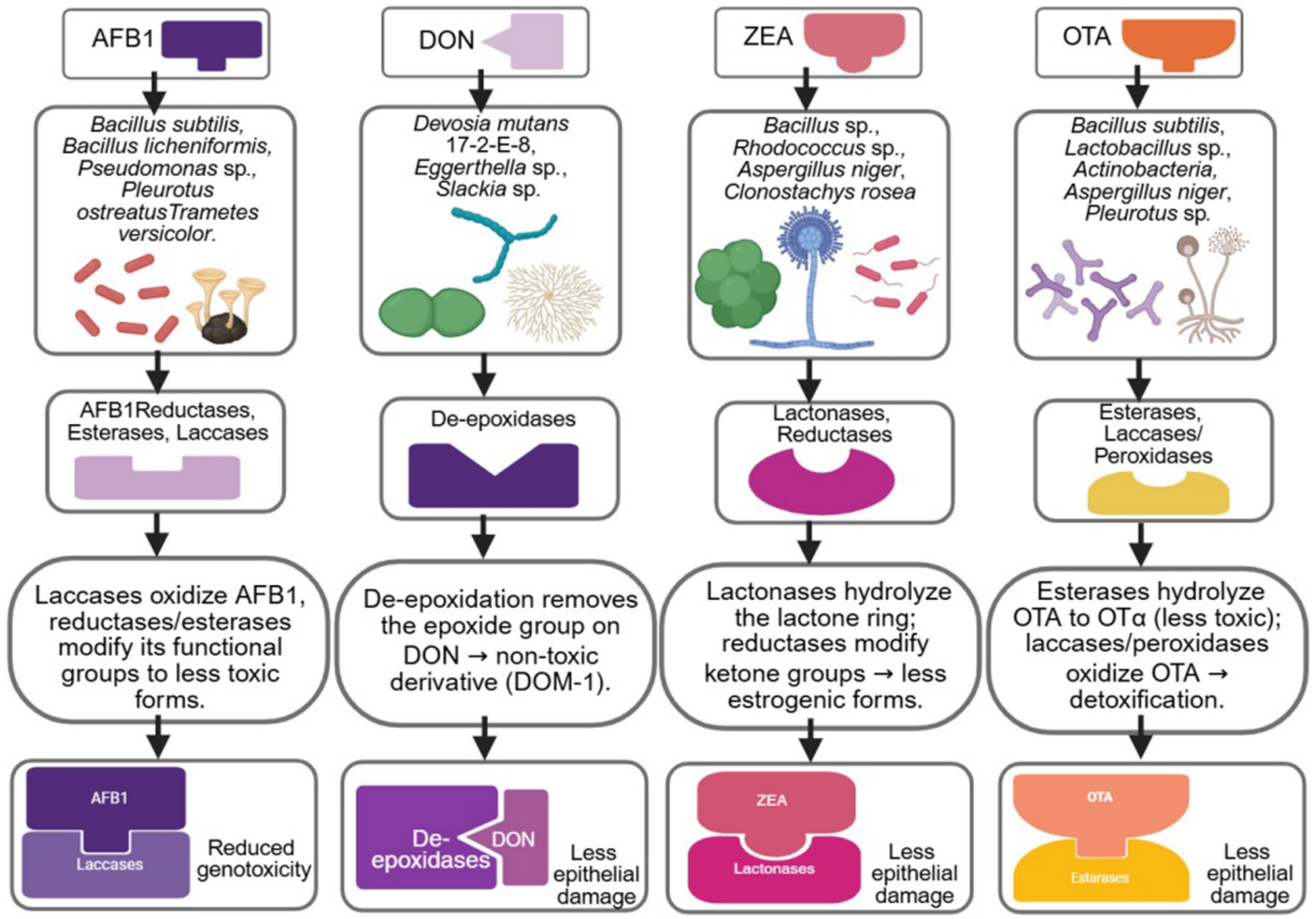
Microbial enzymatic detoxification pathways for AFB1, DON, ZEA, and OTA. Created in BioRender. Busselberg, D. (2025). https://app.biorender.com/illustrations/69285607360c281b12d4a66b. Arrows indicate mechanistic associations rather than sequential reactions; dashed arrows represent indirect or strain-dependent pathways. *In vitro*.

Microorganisms utilize diverse enzymatic systems to modify and neutralize mycotoxins within the gastrointestinal tract (39, 40). A key pathway is the de-epoxidation of DON, which converts the reactive epoxide group into a less toxic derivative (41, 42). Detoxification of ZEA frequently occurs through lactonase-mediated ring opening, reducing its estrogenic activity (43, 44). Additionally, mycotoxin-reducing enzymes such as reductases active against aflatoxins and related compounds, as well as esterases, laccases, and peroxidases, mediate the structural degradation of mycotoxins such as AFB, ZEA, DON, OTA, PAT, and FB, produced by fungi like *Aspergillus flavus* and *A. parasiticus*. Biological and enzymatic detoxification is preferred over physical or chemical methods due to its selectivity, environmental safety, and preservation of food and feed quality (45–47). These enzymatic activities vary widely between microbial species and even among strains, leading to significant differences in detoxification efficiency (40, 45, 48). As a result, specific probiotic strains such as *Lactobacillus* sp., *Bifidobacterium* sp., and *Bacillus* sp. demonstrate enhanced catalytic potential, making them promising candidates for targeted mycotoxin mitigation (46, 47, 49). Beyond lactic acid bacteria, emerging evidence highlights the role of non-conventional gut-associated taxa. Notably, *Devosia* species have been shown to enzymatically transform specific mycotoxins, such as DON, into less toxic derivatives through defined reductive and hydrolytic pathways mediated by enzymes including G15-DDH, G15-AKR1, and G15-AKR6, with PQQ synthesis supporting their activity (50). This expands the field of mycotoxin detoxification beyond classic probiotics and supports omics-guided identification of next-generation detoxifying strains. Figure 2 illustrates the microbial enzymatic pathways involved in the detoxification of AFB1, DON, ZEA, and OTA.

#### 3.3.1 Toxicological relevance of microbial metabolites

While microbial metabolism can reduce toxicity, it does not invariably result in detoxification. Certain microbial biotransformations can produce metabolites with retained or enhanced biological activity, reflecting the strain- and enzyme-specific nature of mycotoxin degradation, where isolated microbial enzymes often exhibit higher substrate specificity and distinct positional or chiral selectivity compared to whole-cell systems (51). For example, ZEA conversion to *α*-zearalenol by some lactonase-producing bacteria can maintain or increase estrogenicity. Similarly, de-epoxidation of DON by bacteria such as *Eggerthella* sp. DII-9 generates metabolites that may retain biological activity, highlighting that microbial biotransformation is not always equivalent to detoxification (52). OTA degradation products remain incompletely characterized, and their toxicological significance is uncertain, despite OTA’s well-documented teratogenic, immunotoxic, genotoxic, carcinogenic, neurotoxic, and hepatotoxic effects in humans and livestock and its widespread occurrence in foods such as cereals, dried fruits, coffee, and wine (53). These examples indicate the importance of strain-specific assessment in microbial detoxification strategies, illustrating that the safety and efficacy of probiotic or gut-associated detoxifying microbes must be carefully evaluated. Collectively, this reinforces the need for omics-guided screening and functional characterization to identify strains that reliably produce non-toxic metabolites, while avoiding the unintended generation of harmful intermediates.

### 3.4 Probiotic detoxification strategies

Probiotics counteract mycotoxins through a combination of physical, biochemical, and ecological mechanisms (54). One major approach is cell-wall binding, in which peptidoglycan, teichoic acids, and other structural polymers bind toxins, thereby reducing their intestinal absorption. This binding effect has been demonstrated specifically for ZEA and AFB1 (55, 56). This is complemented by adsorption and sequestration processes that immobilize toxins within the gut (57). Specific probiotic strains, including *Lactobacillus* sp., *Pediococcus* sp., and *Bacillus* species, have been shown to enzymatically biotransform AFB1 into less harmful metabolites (58). Beyond direct detoxification, probiotics exert protective roles by outcompeting pathogenic microorganisms, limiting dysbiosis-associated toxicity, and strengthening host defenses (53). They enhance mucosal immunity, stimulate epithelial regeneration, and support intestinal barrier integrity. Specific evidence supports *Lactobacillus rhamnosus* GG for AFB1 binding and *Lactobacillus plantarum* strains for ZEA and DON detoxification. Table 1 presents various probiotic strains and outlines their specific mechanisms for detoxifying mycotoxins.

### 3.5 Effects on cancer pathways

**Figure 3:**
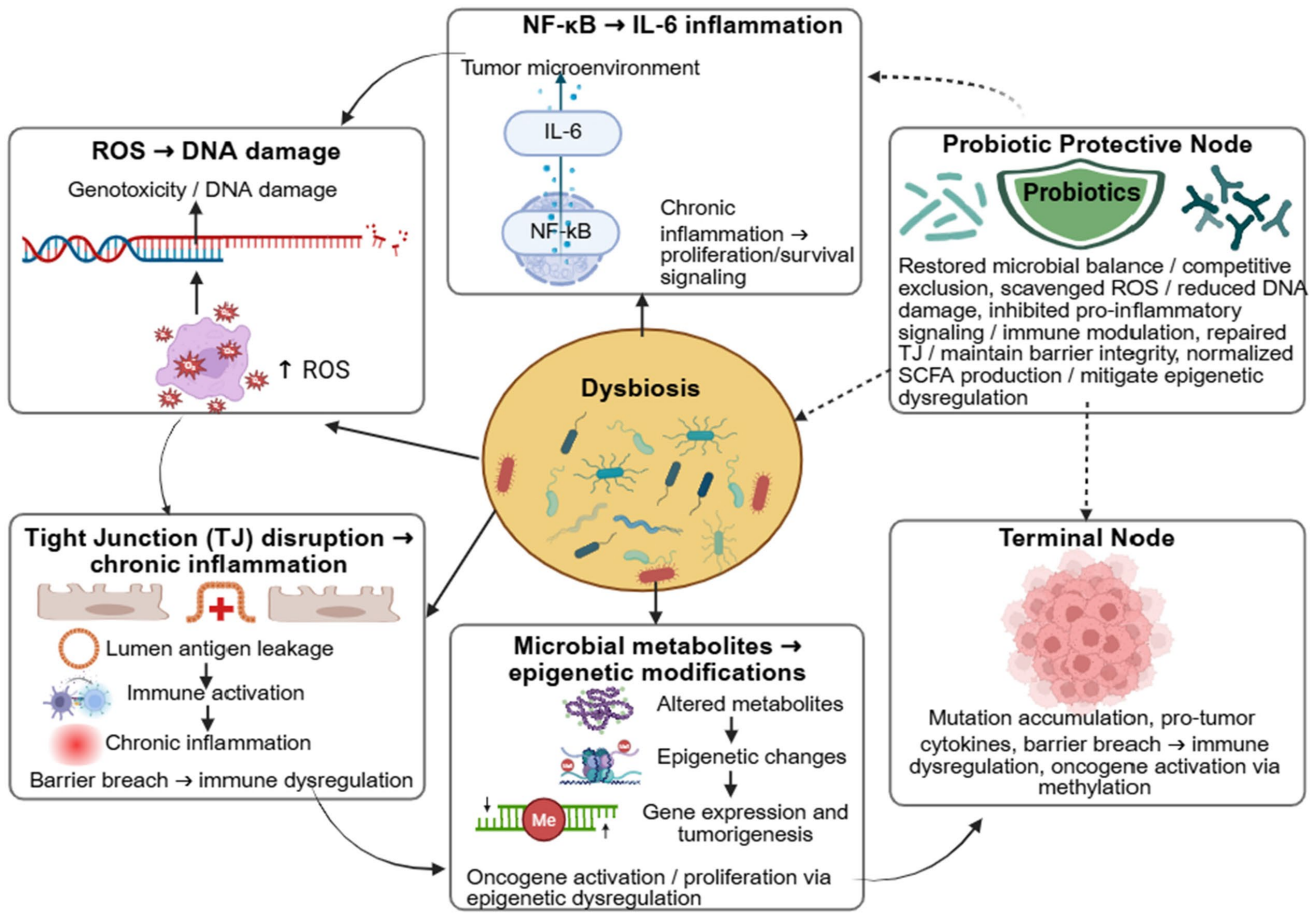
Mechanistic crosstalk between mycotoxin-induced dysbiosis, cancer, and protective probiotic strategies. Created in BioRender. Busselberg, D. (2025). https://app.biorender.com/illustrations/692ac77dfef285debdb7802c.

Mycotoxins are implicated in multiple molecular pathways associated with cancer development. Specifically, toxins such as T-2, DON, ZEA, AFB1, and OTA trigger pro-inflammatory signaling pathways, including NF-κB, IL-6, and TNF-*α*, creating a sustained inflammatory environment that promotes tumor initiation and progression (54, 59). Specific mycotoxins such as AFB1, ZEA, OTA, and DON have been reported to induce genotoxic effects, including DNA adduct formation (AFB1, ZEA), chromosomal abnormalities (DON, ZEA), and disruption of DNA repair pathways (OTA, AFB1), potentially contributing to carcinogenesis and impaired cellular homeostasis (57, 60). Increasing evidence indicates that interactions between specific mycotoxins, such as AFB1, FB1, DON, and ZEA, and the gut microbiome can drive epigenetic modifications, including altered DNA methylation and microRNA expression, which further shape the host’s cancer risk (58, 61, 62). Moreover, exposure to these mycotoxins can induce microbial dysbiosis and may promote the expansion of pro-carcinogenic microbial populations and the production of harmful metabolites, thereby amplifying pathways that support tumorigenesis. DON and ZEA are gut-active mycotoxins that alter microbiota composition, modulate immune responses, and contribute to a tumor-promoting microenvironment. Mechanistic studies in animal models and human cells have demonstrated links between mycotoxin-induced dysbiosis, epigenetic dysregulation, immune modulation, and tumor initiation, particularly in gut-related cancers such as colorectal cancer (CRC) and hepatocellular carcinoma (63–65). While evidence is strongest for these gut-associated cancers, data on the role of mycotoxin–microbiome interactions in other malignancies remain limited. Figure 3 depicts the mechanistic crosstalk between mycotoxin-induced dysbiosis and carcinogenesis.

Beyond direct genotoxicity, the gut microbiome plays a critical intermediary role in modulating cancer risk by shaping inflammatory tone, epithelial integrity, and host epigenetic regulation, with age-related physiological changes further influencing microbial composition, DNA methylation and histone modification patterns, immune competence, and host–microbe–drug interactions that collectively affect cancer susceptibility and therapeutic outcomes (66). Mycotoxin-induced dysbiosis is frequently associated with reduced production of short-chain fatty acids (SCFAs), particularly butyrate, which normally exerts anti-carcinogenic effects through histone deacetylase (HDAC) inhibition, reinforcement of epithelial barrier function, and suppression of pro-inflammatory signaling, processes that are strongly influenced by dietary patterns, as fibre-rich diets and probiotic interventions can promote SCFA-producing commensals, partially restore dysbiosis, and enhance butyrate-mediated epigenetic and immunomodulatory protection (67). Reduced SCFA availability has been linked to aberrant epigenetic programming, increased intestinal permeability, and enhanced activation of oncogenic pathways such as NF-κB and STAT3, thereby facilitating chronic inflammation–driven tumorigenesis, particularly in colorectal cancer, where diminished luminal SCFAs impair epithelial barrier function and immune regulation while promoting epigenetic alterations that support tumor initiation and progression (68). Dysbiotic microbiota may also amplify IL-6–mediated signaling, which promotes cancer cell survival, angiogenesis, and immune evasion, particularly in inflammation-associated cancers, by reshaping the tumor microenvironment through sustained inflammatory cytokine production, immunometabolic reprogramming, and impaired anti-tumor immune surveillance (69).

Specific mycotoxin–cancer associations further illustrate the microbiome’s modulatory role. AFB1 is a well-established risk factor for hepatocellular carcinoma (HCC), particularly in Asia and sub-Saharan Africa where dietary exposure is high, and emerging evidence suggests that gut microbial composition influences AFB1 bioactivation and detoxification, with dysbiosis altering bacterial–xenobiotic interactions that affect epoxide formation, DNA adduct burden, and hepatic cancer risk; similar microbiome-mediated perturbations have been reported for other carcinogenic mycotoxins, including FBs, OTA, ZEA, and trichothecenes, which disrupt intestinal homeostasis and contribute to hepatocarcinogenesis (5, 70). Certain microbial enzymes and microbial–host interactions can modulate cytochrome P450–dependent conversion of AFB1 into its highly genotoxic epoxide form, thereby influencing hepatic DNA adduct burden and cancer susceptibility, by regulating the activity and expression of key bioactivating enzymes, including CYP1A1/2, CYP2A6, and CYP3A4, which catalyze the formation of aflatoxin B1-8,9-epoxide (AFBO), while microbial-mediated shifts in xenobiotic metabolism may attenuate or exacerbate this bioactivation process (71). Conversely, Beneficial microbes, including probiotic lactic acid bacteria (*Lactobacillus rhamnosus* GG, *L. rhamnosus* LC705, *Lactobacillus casei* Shirota, *Lactococcus lactis*, and selected *Bifidobacterium* species), can reduce systemic exposure to AFB1 through cell wall adsorption, microbiota modulation, and barrier reinforcement, while biotransforming microbes such as *Bacillus subtilis* detoxify AFB1 into less harmful metabolites (e.g., aflatoxin D1), thereby attenuating hepatic DNA damage and carcinogenic risk (54, 72). In the gut, trichothecenes such as DON and T-2 toxin contribute to colorectal cancer risk by disrupting tight junctions and epithelial integrity, thinning the mucus layer, altering immune and inflammatory responses, and perturbing microbial homeostasis; these combined effects compromise the physical, chemical, immunological, and microbial barriers of the intestine, sustaining low-grade inflammation and creating a permissive environment for tumor initiation and progression (73). Microbiome-mediated alterations in immune surveillance and metabolite profiles further exacerbate these effects, linking chronic mycotoxin exposure to inflammation-driven colorectal carcinogenesis.

Microbial enzymatic activity and host–microbe interactions critically determine the carcinogenic potential of mycotoxins which include AFB1, FBs, OTA, ZEA, and trichothecenes (DON, T-2 toxin). Microbial modulation of cytochrome P450 enzymes (CYP1A1/2, CYP2A6, CYP3A4) regulates the formation of AFB1-8,9-epoxide (AFBO), influencing hepatic DNA adducts and hepatocellular carcinoma risk, while probiotic lactic acid bacteria (*Lactobacillus rhamnosus* GG, *L. rhamnosus* LC705, *Lactobacillus casei* Shirota, *Lactococcus lactis*, selected *Bifidobacterium* species) and *Bacillus subtilis* detoxify AFB1 and support microbial balance. Beyond direct genotoxicity, mycotoxin-induced dysbiosis diminishes short-chain fatty acid (SCFA) production, disrupts epithelial and immune homeostasis, and alters epigenetic regulation, activating oncogenic pathways such as NF-κB, STAT3, and IL-6. These perturbations promote chronic inflammation–driven tumorigenesis, particularly in colorectal cancer, where impaired SCFA-mediated barrier and epigenetic signaling favor tumor initiation and progression. Age-related microbiome shifts and host–microbe–drug interactions further modulate susceptibility and therapeutic outcomes, highlighting the microbiome as an active mediator of mycotoxin-driven cancer and a potential target for intervention.

### 3.6 Innovations and technological advances

Technological advancements are offering new solutions to improve mycotoxin detoxification and protect gut health (74, 75). Genetically engineered probiotics capable of producing higher levels of detoxification enzymes such as aflatoxin-detoxifizyme, epoxide hydrolase, and esterase provide improved degradation of harmful mycotoxins, such as AFB1, OTA, and ZEA (76, 77). Using CRISPR-based editing, microbial strains can be precisely modified to enhance safety, metabolic activity, and specificity toward targeted toxins (78–80). For instance, CRISPR-edited *Lactobacillus* strains expressing enhanced de-epoxidase activity have been proposed for DON detoxification, while nanoencapsulation of *Saccharomyces boulardii* improves the stability of OTA binding. The development of multispecies synbiotic formulations integrates complementary microbial functions with selective prebiotic substrates, resulting in more robust and mechanistically supported detoxification and anti-inflammatory outcomes. For example, a recent randomized, placebo-controlled clinical trial demonstrated that a 24-strain multispecies synbiotic combined with a polyphenol-based prebiotic significantly enhanced gut microbial diversity, increased production of urolithin A and butyrate, and reduced systemic inflammation in healthy adults. These metabolites are known to modulate immune signaling, suppress pro-inflammatory cytokine release, and reinforce epithelial and blood–brain barrier integrity. Such mechanisms are particularly relevant in neuroinflammatory conditions, including Zika virus infection, in which microglial activation, oxidative stress, and the release of inflammatory mediators contribute to neuronal death and altered neuron–glia interactions. These synbiotic effects would be beneficial against AFB1 and FB1 which are the most prominent gut-active mycotoxins known to induce microbial dysbiosis, systemic inflammation, epithelial barrier disruption, and mitochondrial dysfunction, as well as to exacerbate oxidative stress and pro-apoptotic pathways in host cells, thereby increasing susceptibility to toxin-induced inflammatory cascades. By promoting short-chain fatty acid production and polyphenol-derived metabolites with immunoregulatory properties, multispecies synbiotics provide a biologically plausible, cost-effective strategy to attenuate toxin- or virus-induced inflammatory cascades and support host detoxification pathways (66, 67). In parallel, nanoencapsulation systems are increasingly being used to ensure targeted, protected delivery of probiotics and bioactive compounds to the gastrointestinal tract. Established approaches include the use of nanoemulsions and polymer-based micro- and nano-capsules derived from natural biopolymers (e.g., alginate, chitosan, and starch derivatives), which have been shown to enhance probiotic survival during processing, storage, and gastrointestinal transit. These systems protect probiotic cells and prebiotic compounds, such as inulin and polyphenols, from harsh environmental conditions, including low pH, bile salts, and temperature fluctuations, while enabling controlled release at the site of action in the intestine. In non-dairy functional food matrices, nano-encapsulation has been successfully employed to improve probiotic viability, preserve sensory quality, and increase the bioavailability of health-promoting metabolites. Furthermore, integrating nanotechnology into symbiotic formulations supports selective microbial stimulation and sustained metabolite production, aligning with emerging precision nutrition strategies aimed at improving digestive, immune, metabolic, and neurocognitive health (68–70). Furthermore, omics technologies such as metagenomics, proteomics, and metabolomics are enabling high-resolution mapping of microbial–toxin interactions, thereby guiding the rational design of next-generation detoxification strategies. For example, integrated metagenomic and transcriptomic analyses have been successfully applied to hybrid algal–bacterial systems to elucidate shifts in microbial community structure, functional gene expression, and metabolic pathways associated with enhanced removal of organic pollutants, heavy metals, nutrients, and contaminants of emerging concern. Similarly, in the context of gut health, omics-driven approaches are increasingly used to characterize how probiotic, prebiotic, synbiotic, and postbiotic interventions modulate microbial metabolism, inflammatory signaling, and host–microbe interactions. Recent advances in micro- and nanoencapsulation of biotics further benefit from these technologies by enabling precise evaluation of microbial viability, metabolic output, and controlled release behavior within the gastrointestinal tract. Collectively, the convergence of omics technologies with encapsulation and microbial engineering provides a robust framework for developing targeted, mechanism-informed detoxification and disease-prevention strategies (71, 72).

### 3.7 Challenges and limitations in current research

Although the role of microbes in mycotoxin detoxification is increasingly recognized, several limitations still hinder advancement in this area (73, 81). One key challenge is the wide variability in strain performance, as the detoxifying capacity of probiotics and other beneficial microbes can differ significantly among species, subspecies, and even individual isolates (82–84). The lack of standardized methods for assessing detoxification further complicates progress, making it difficult to compare results across studies or reliably determine the actual effectiveness of specific strains (85, 86). Moreover, inconsistencies between *in vivo* and *in vivo* findings present another obstacle, since microbial activity observed under controlled laboratory conditions often fails to mirror the complex, dynamic environment of the human gastrointestinal tract (87–89). Safety concerns also remain essential, particularly for immunocompromised individuals who may be vulnerable to infections or unintended immune reactions when exposed to live microbial agents (90). Additionally, the natural diversity of human gut microbiomes creates further complexity, as differences in baseline microbial composition can influence detoxification efficiency, treatment outcomes, and overall host response (91, 92).

### 3.8 Regulatory perspectives

The regulatory framework governing mycotoxin control and microbial-based interventions is continually evolving (93, 94). Many countries enforce strict permissible limits for major mycotoxins such as AFs, FBs, OTA, and ZEA to ensure food safety and protect public health (95). Regulatory bodies also distinguish between probiotics used as food supplements and those intended for therapeutic purposes, with therapeutic strains subject to more stringent safety, efficacy, and quality evaluations (96–98). The development of engineered or genetically modified microbial strains introduces additional regulatory challenges, including considerations of biosafety, environmental impact, and consumer acceptance (99, 100). These factors underscore the need for harmonized regulations and more straightforward guidelines to facilitate the safe development, approval, and commercialization of innovative microbial solutions for mycotoxin mitigation. Table 3 provides an overview of global regulatory limits for major mycotoxins and the current status of probiotic-based interventions.

**Table 3:** Global regulatory limits for major mycotoxins and status of probiotic-based interventions.

Mycotoxin	Typical international limits (Codex/EFSA/FDA)	Jurisdictions with stricter limits	Approved/Recognized probiotic applications (for mycotoxin mitigation)	Main regulatory barriers	Reference
AFs (AFB₁ and total AFs)	Codex: commodity-specific MLs. EU: very low MLs for many nuts and cereals. FDA: action levels (e.g., AFM₁ in milk).	EU and high-income countries (e.g., Japan)	None approved for detoxification claims; probiotics allowed as foods/supplements only	Claim restrictions; strain-specific GRAS/QPS; proof of ML reduction required; human vs. feed limits differ; clinical validation needed for human detox claims.	(180–182)
DON	Codex: guidance for cereals/infant foods. EU: tightened MLs (Regs. 2023/915; 2024/1022). FDA: guidance/action levels.	EU and selected trading partners	None approved; evidence mainly *in vitro*/animal studies.	Lack of field/clinical validation; novel food/additive approval needed; human supplement detox claims require clinical trials; feed approval pathways differ.	(183, 184)
ZEA	Codex/EFSA/FDA: commodity-specific MLs/guidance for food and feed.	EU and countries with conservative feed limits.	None approved; experimental binding/degradation by LAB, Bacillus, yeasts.	Strain variability; matrix effects; reversible binding; human supplement claims require safety/efficacy demonstration; feed rules differ.	(185, 186)
OTA	Codex/EFSA/FDA: low μg/kg MLs for cereals, coffee, dried fruits.	EU and coffee-importing countries	None approved; yeast and LAB adsorption shown experimentally.	OTA stability; limited efficacy in complex foods; detoxification claims for human supplements largely unapproved; feed risk assessment differs.	(187–189)
FBs (FB₁ + FB₂)	Codex/EFSA/FDA: MLs/guidance mainly for maize and maize products.	EU and maize-dependent markets	None approved; microbial/enzymatic degradation explored mainly for feed.	Feed-additive authorization; GMO/novel-feed regulations; human probiotic detox claims unapproved; clinical evidence required	(156, 190, 191)

AFs, aflatoxins; DON, deoxynivalenol; ZEA, zearalenone; OTA, ochratoxin A; FBs, fumonisins, MLs, maximum limits; EFSA, European Food Safety Authority; FDA, U.S. Food and Drug Administration. “Approved applications” refers to regulatory authorisation for explicit mycotoxin detoxification claims, not general probiotic use.

## 4 Innovations, challenges, limitations, and future work

### 4.1 Innovations

Advances in microbiome research have introduced several innovative approaches for enhancing detoxification processes and overall host health (101, 102). Next-generation probiotics (NGPs) are newly characterized microbial species with targeted therapeutic functions beyond those of conventional probiotics, developed for both nutritional and pharmaceutical applications. Representative candidates include *Akkermansia muciniphila*, *Faecalibacterium prausnitzii*, and selected *Bacteroides* and *Clostridium* species, which have demonstrated the ability to improve gut barrier integrity, produce beneficial metabolites, such as short-chain fatty acids, and modulate immune and metabolic pathways relevant to inflammatory, metabolic, and neuroinflammatory disorders. Recent advances in synthetic biology and CRISPR-based engineering have further enabled the development of NGPs with enhanced colonization capacity, metabolic activity, and targeted bioactive delivery, supporting personalized microbiome-based interventions (90, 103, 104). Microbiome engineering extends these innovations by enabling deliberate modification of microbial communities or genomes to optimize functional traits. Engineered strains and consortia have been designed to enhance detoxification capacity and therapeutic precision, with applications ranging from chronic disease management to environmental bioremediation of heavy metals and complex organic pollutants. These strategies combine community-level manipulation with strain-level engineering to achieve controlled and stable functional outputs across diverse biological contexts (105, 106). Emerging intelligent gut biosensors further complement these approaches by enabling real-time monitoring of microbial activity and toxin-related signals within the gastrointestinal tract. Ingestible biosensors based on engineered microbes or enzyme-driven systems have been developed to detect pH changes, metabolites, biomarkers, and specific toxins under harsh gastrointestinal conditions. Such platforms support early disease detection, treatment monitoring, and real-time assessment of probiotic or detoxification efficacy, with ongoing advances in biomaterials and device miniaturization improving biocompatibility and performance (95, 107, 108). Finally, the integration of multi-omics platforms such as genomics, transcriptomics, proteomics, and metabolomics, provides system-level insights into host–microbe interactions and microbial functionality. Genome-scale metabolic models and omics-guided analyses have been applied to predict microbial contributions to host metabolism, guide probiotic design, and optimize plant growth–promoting microbial consortia by identifying key traits involved in nutrient acquisition, stress tolerance, and signaling networks (109–111). Collectively, these innovations offer a data-driven framework for advancing detoxification strategies, precision monitoring, and microbiome-informed health interventions.

### 4.2 Challenges

Despite advances in microbial-based strategies, several obstacles continue to limit their translation into practical applications. Strain instability remains a key concern, as the viability, functional traits, and detoxification capacity of beneficial microbes may diminish during storage, processing, or passage through the gastrointestinal tract, with *Lactobacillus rhamnosus* which has been reported to be particularly sensitive to acid, oxygen, and heat stress during storage and processing, which can reduce its survival and functional activity, *Bifidobacterium longum* which is primarily sensitive to oxygen exposure and the conditions encountered during gastrointestinal transit, affecting its ability to reach the colon in sufficient numbers to exert beneficial effects and *Enterococcus durans* which has been observed specifically during interactions with therapeutic treatments, and may exhibit instability (112, 113). Human clinical evidence is still limited, restricting robust conclusions about the efficacy, safety, and long-term impact of these interventions in real-world conditions (114, 115). Additionally, the broader physiological consequences, particularly regarding cancer-related outcomes, remain poorly understood, with insufficient data on chronic exposure, immune interactions, and potential unintended effects (116, 117). Regulatory and standardization gaps further constrain implementation, as variability in probiotic formulations, dosing, and quality control can influence effectiveness. Addressing these challenges will require targeted experimental studies, well-designed clinical trials, and cohort studies that assess the resistance, viability, and functionality of probiotic strains under storage, processing, and gastrointestinal conditions. Observational and *in vivo* approaches can complement these trials to provide mechanistic insights while bypassing some ethical constraints. Standardized research frameworks and cautious interpretation of findings will be essential to ensure safe and evidence-based application of probiotics in humans.

### 4.3 Limitations

Despite growing interest in microbial detoxification, several limitations restrict current progress. Mechanistic understanding of microbial mycotoxin degradation remains incomplete for many compounds, making precise characterization of detoxification pathways difficult (118, 119). Most evidence derives from *in vitro* or animal studies, highlighting notable gaps in human-based data and limiting direct translational applicability. Although biological strategies, including probiotics and microbial enzyme-based approaches, have demonstrated *in vitro* promising efficacy *in vitro* and in animal models for degrading mycotoxins, their effectiveness and safety in humans remain largely untested. Furthermore, reliance on animal models is limited, as they often poorly predict human toxicity, highlighting the need for human-focused studies to validate these interventions (120, 121). An important limitation insufficiently addressed in current research is the mismatch between experimental exposure models and real-world dietary exposure scenarios. Human populations are typically exposed to chronic low-dose mycotoxin intake rather than acute high-dose exposure, and co-exposure to multiple mycotoxins (e.g., AFB1 with FBI, or DON with ZEA) is common in contaminated staple foods. Such combined exposures may produce additive or synergistic effects on gut barrier integrity, immune activation, and microbial dysbiosis, which are rarely captured in controlled experimental designs. Methodological limitations also exist in the detection and quantification of mycotoxins in biological matrices relevant to probiotic efficacy. Current analytical approaches often lack sufficient sensitivity or validation for measuring low mycotoxin concentrations in feces, intestinal tissues, or luminal contents, making it difficult to accurately assess microbial biotransformation, toxin sequestration, or reduced intestinal uptake. This analytical gap limits the ability to directly link probiotic intervention to biologically meaningful reductions in exposure under realistic dietary conditions. In addition, the *in vivo* bioavailability, gastrointestinal survival, colonization efficiency, and functional metabolic activity of probiotics remain highly variable and strain dependent. Factors such as gastric acidity, bile exposure, host diet, baseline microbiome composition, and inter-individual variability can significantly influence probiotic performance, potentially limiting reproducibility and clinical efficacy. Methodological variability, including differences in experimental design, assay conditions, microbial strain characterization, and analytical techniques, further complicates comparisons across studies (122, 123). Taken together, these limitations emphasize the need for unified, clinically oriented, and mechanistically informed research approaches. Future research should prioritize well-designed human studies, such as controlled dietary interventions assessing the impact of probiotic supplementation on biomarkers of AFB1 or ZEA exposure. Where direct exposure is ethically constrained, observational cohort studies in populations with naturally varying mycotoxin intake, combined with mechanistic biomarkers, can provide complementary evidence on safety and efficacy in real-world settings.

### 4.4 Future research directions

Moving forward, several strategic areas warrant emphasis to strengthen this field. Well-designed human clinical trials are needed to validate microbial detoxification enzymes and probiotic interventions, providing robust evidence of safety and practical effectiveness under real-world conditions (124–126). Based on the current body of evidence synthesized in this review, AFB1, DON, ZEA, OTA, and FB1 emerge as priority mycotoxins for future investigation, given their strong associations with gut dysbiosis, epithelial barrier disruption, inflammation, and carcinogenic signaling. Among these, AFB1 and ZEA show the most consistent and mechanistically supported potential for probiotic intervention. Multiple *Lactobacillus* sp. and *Bifidobacterium* strains have demonstrated effective binding, enzymatic biotransformation, or both, reducing intestinal uptake and systemic bioavailability. DON also represents a high-priority target, particularly through microbial de-epoxidation pathways that attenuate its ribotoxic and epithelial-damaging effects. Although evidence for OTA and FB1 detoxification remains more limited, emerging data suggest that selected probiotic strains and multispecies consortia may partially mitigate their intestinal absorption and inflammatory consequences, warranting further focused investigation. Screening and characterization of native microbial strains from fermented foods may reveal candidates with high detoxifying potential, yet their efficacy must be confirmed across diverse populations and dietary contexts (127–129). Artificial intelligence and computational modeling offer opportunities to predict interactions between toxins and the microbiome. These approaches leverage machine learning (ML) and deep learning algorithms, convolutional neural networks (CNNs), recurrent neural networks (RNNs), and graph neural networks (GNNs), to analyze high-throughput microbiome datasets from metagenomics, metabolomics, transcriptomics, and proteomics. AI-driven analysis can identify microbial species, functional genes, and metabolic pathways, and predict host responses to toxins or probiotic interventions. These approaches leverage machine learning and deep learning algorithms to analyze high-throughput microbiome datasets and may help prioritize strain–toxin pairings for AFB1, DON, and ZEA based on predicted binding affinity, enzymatic compatibility, and host-response modulation. However, these computational predictions require careful experimental validation and integration with empirical data to ensure accuracy, reliability, and translational relevance (130, 131). The development of therapeutic microbial consortia, where multiple strains act synergistically, presents another promising avenue, particularly for complex exposures involving co-occurring mycotoxins such as AFB1–FB1 or DON–ZEA, though standardized evaluation frameworks are essential to ensure reproducibility and translational relevance (132, 133). Finally, strengthening regulatory pathways and quality control standards will be critical to support safe, evidence-based application of microbial-based strategies in clinical and commercial settings.

## 5 Conclusion

This review provides a detailed, mechanistic overview of microbiome–mycotoxin interactions, illustrating how mycotoxins disrupt gut microbial balance and influence pathways linked to cancer development. By focusing on microbial detoxification processes, omics-driven mechanistic insights, and probiotic strategies, the review synthesizes current evidence and clarifies the complex relationships governing gut health, toxin metabolism, and cancer-related pathways. The findings highlight the potential of probiotics as supportive strategies for mycotoxin mitigation while also emphasizing critical knowledge gaps that limit direct translation into clinical or regulatory applications. Future research should prioritize long-term human studies to validate experimental observations, assess sustained health effects, and confirm probiotic efficacy across diverse populations and dietary contexts. Improved strain-level characterization and biomarker identification will enhance precision in selecting microbial interventions, while advances in engineered microbial systems may expand the potential for detoxification. The integration of artificial intelligence and computational modeling using ML and deep learning approaches, such as CNNs, RNNs, and GNNs, offers a complementary approach to predicting interactions among toxins, microbial species, and probiotics, though careful experimental validation remains essential. Strengthening and harmonizing regulatory frameworks will also be critical to ensure the safe, standardized, and evidence-based application of microbial-based interventions.

## Glossary

AFM: Aflatoxins M1
AFB1: Aflatoxin B1
CRC: Colorectal Cancer
CRISPR: Clustered Regularly Interspaced Short Palindromic Repeats
CYP450: Cytochrome P450
DNA: Deoxyribonucleic Acid
DON: Deoxynivalenol
EFSA: European Food Safety Authority
EU: European Union
FDA: Food and Drug Administration
FB1: Fumonisin B1
GRAS: Generally Recognized as Safe
IL-6: Interleukin-6
LAB: Lactic Acid Bacteria
LPS: Lipopolysaccharide
MLs: Maximum Levels
NGP: Next-Generation Probiotics
NF-κB: Nuclear Factor kappa-light-chain-enhancer of activated B cells
OTA: Ochratoxin A
Patulin: Patulin
PubMed: Public/Publisher MEDLINE database
QPS: Qualified Presumption of Safety
ROS: Reactive Oxygen Species
SciELO: Scientific Electronic Library Online
T-2: Toxin T-2 Toxin.
TNF-*α*: Tumor Necrosis Factor alpha
ZEA: Zearalenone.
microRNA: Micro-Ribonucleic Acid
