# Supplementary material for: Microbiome–mycotoxin interactions and probiotic strategies: implications for gut health and cancer
Source: Front Nutr. 2026 Feb 25;13:1783295. doi: 10.3389/fnut.2026.1783295 (PMC12975935; doi:10.3389/fnut.2026.1783295)
Supplement: Supplementary file 1 [file Table_1.docx]

**Supplementary Table S1.** Database-specific search strategies and quantitative outputs for the structured narrative literature synthesis

| **Database** | **Records Retrieved** | **After Duplicates** | **Screened** | **Full Texts Assessed** | **Included** |
| --- | --- | --- | --- | --- | --- |
| PubMed | ~780 | ~520 | ~520 | 110 | 72 |
| ScienceDirect | ~1,120 | ~680 | ~680 | 95 | 64 |
| Google Scholar | ~900 | ~450 | ~450 | 55 | 63 |
| Total | ~2,800 | ~1,650 | ~1,650 | ~260 | 199 |
