# Supplementary material for: Microbiome–mycotoxin interactions and probiotic strategies: implications for gut health and cancer
Source: Front Nutr. 2026 Feb 25;13:1783295. doi: 10.3389/fnut.2026.1783295 (PMC12975935; doi:10.3389/fnut.2026.1783295)
Supplement: Supplementary file 2 [file Table_2.docx]

**Supplementary Table S2**. Operational Framework for Indicative Strength of Evidence

| Strength Level | Criteria | Examples from Table 2 |
| --- | --- | --- |
| Strong | ≥2 in vivo studies and a consistent detoxification mechanism (binding, enzymatic degradation, or immune modulation) | *Lactobacillus* *rhamnosus* GG, *Saccharomyces* *cerevisiae* / *S*. *boulardii* |
| Moderate | Multiple in vitro studies with limited in vivo support | *Lactobacillus* *plantarum*, *Lactobacillus* *acidophilus*, *Lactobacillus* *casei*, *Bifidobacterium* *longum*, *Bacillus* *subtilis* |
| Limited | In vitro only or inconsistent results across strains/mechanisms | *Lactococcus* *lactis*, *Bifidobacterium* *bifidum*, *Enterococcus* *faecium* |
| Emerging / Variable | Preliminary evidence from commercial blends or newly tested strains; inconsistent or partial mechanistic data | *Pediococcus* *pentosaceus*, mixed commercial probiotic consortia |

Note:

1. Consistency refers to the reproducibility of detoxification effects across independent studies.
2. In vivo refers to animal studies assessing mycotoxin reduction or host health outcomes.
3. Clinical refers to human studies on probiotic safety and efficacy.
4. This framework ensures transparency and reproducibility
